# Simulation Analysis and Process Evaluation of Cooling Hole Forming Precision in Mask Assisted Electrochemical Machining Based on GH4169

**DOI:** 10.3390/ma15051973

**Published:** 2022-03-07

**Authors:** Zhaolong Li, Ye Dai

**Affiliations:** Key Laboratory of Advanced Manufacturing Intelligent Technology of Ministry of Education, Harbin University of Science and Technology, Harbin 150080, China; lizhaolong@hrbust.edu.cn

**Keywords:** electrochemical micro machining of titanium alloy, manufacturing process, shape accuracy, modeling, mask electrochemical machining, influence of tool electrode on titanium alloy

## Abstract

Good heat dissipation performance of aero-engine an effectively improve the service performance and service life of aero-engine. Therefore, this paper studies the machining method of cooling holes of high-temperature existent material GH 4169 for aero-engine innovatively puts forward the mask electrochemical machining method of cooling holes and explores the entrance morphology and taper formation law of the hole structure of high-temperature resistant material GH 4169. The mathematical model of anode dissolution of cooling holes in ECM is established, and the influence of voltage and electrolyte flow rate on cooling holes in ECM is analyzed. Compared with the mask-less electrochemical machining, the inlet radius of cooling holes in mask electrochemical machining is reduced by about 16.0% and the taper is reduced by 52.8% under the same machining parameters, which indicates that the electrochemical machining efficiency of mask is higher and the machining accuracy is better. Experiments show that the diameter of the mask structure improves the accuracy of the inlet profile of the cooling hole in the ECM. The diameter of the mask increases from 2 mm to 2.8 mm, and the inlet radius of the cooling hole increased from 1.257 mm to 1.451 mm When the diameter of the mask is 2.2 mm, the taper of the cooling hole decreased by 53.4%. The improvement effect is best, and the thickness of the mask has little influence on the forming accuracy of the cooling hole.

## 1. Introduction

With the rapid development of aerospace, weapons, automobiles, medical and other fields, the development trend of mechanical parts is miniaturization and precision. Turbine blade is an important part of the aero-engine, and its structure and process in technology directly affect the performance of the engine. In order to improve the power of the turbine engine, it is necessary to ensure that the turbine works in the high-pressure gas above 1 MPa and the high-temperature environment of 1000 degrees Celsius, which will greatly reduce the service life of turbine blades and lead to the failure of the combustion chamber. Turbine blades are mainly made of high temperature resistant alloys, such asGH 4169. GH4169 nickel-based superalloy has good thermal strength, thermal stability and thermal fatigue, and has become an essential alloy material for manufacturing heat-resistant, corrosion-resistant and impact-resistant parts such as disks and blades of aero-engines and various gas turbines [1].

Machining cooling holes on high temperature resistant alloys can effectively improve the performance and service life of aero-engine [2]. Generally speaking, the machining methods of cooling holes include EDM, laser machining and traditional machining methods such as milling, drilling and hinge. A high-frequency pulse power generator of EDM was developed by Li, C.J. et al., to find the influence of processing speed and the recast layer thickness [3]. Zheng, H. et al. Substrate temperature was observed to affect the machined hole depth and diameter during Ti: sapphire femtosecond laser machining of a copper substrate [4]. Liu, H. et al. Percussion drilling was presented using picosecond ultrashort pulse laser in order to explore processing of deep holes in Ni-based superalloy, ceramic TBCs, and ceramic TBCs/substrate multilayer material. This research has potential applications to blade film cooling holes [5]. Xu, J. et al. hole quality of CFRP/Ti6Al4V stacks using the TiAlN-coated and diamond-coated drills [6]. Iqbal, A. et al. presents a novel technique of cooling only the twist drill between drilling of holes with no effect of the applied cryogenic coolant transferred to the work material [7]. Wang, F. presents the cryogenic cooling milling method can improve milling hole effect and restrict machining defects for AFRP [8]. Zhang, W et al. presents film cooling performance of one row of cylindrical holes integrated with saw-tooth slots was numerically studied at blowing ratios of 0.5, 1.0, 1.5 and 2.0, respectively [9]. Ye, Z. three different cooling conditions were applied to reaming aluminum alloy 7050-T7451 with polycrystalline diamond (PCD) reamers. The results showed that the chip morphology was strongly influenced by the cutting parameters and cooling strategies [10]. As a kind of difficult-to-machine material, GH4169 alloy has the characteristics of poor thermal conductivity, severe work hardening, high affinity and so on, which easily leads to the problems of poor machinability, short service life of cutting tools and poor machined surface quality [11]. The residual stress on the machined surface is the stress that the surface remains and reaches equilibrium when the part is at a constant temperature and is not subjected to external load after cutting [12]. Residual stress on the surface will deform the workpiece and reduce its service life and corrosion resistance. However, hole machining on GH 4169 material by traditional machining method will affect its metal surface performance, the traditional machining requires high hardness of the drill bit, and it is easy to break in the feeding process, so it is difficult to process microstructure on high temperature resistant cemented carbide. In addition, EDM and laser machining are hot machining, which inevitably leads to the formation of hot recast layers and micro cracks on the metal surface. These methods will affect the machining accuracy, stability and working performance of cooling hole. Electrochemical machining utilizes the principle of electrochemical dissolution of anode in electrolyte, and the machined cooling holes has good surface quality, no stress concentration and no surface hardening layer. Therefore, electrochemical machining technology has a broad application prospect in the field of high precision manufacturing such as thin film cooling holes [13].

Electrochemical machining involves many factors, such as electrochemistry, heat transfer, hydrodynamics and so on. Its dissolution and formation state are complex, which cannot be directly determined [14,15]. Many scholars have carried out a lot of simulation and theoretical research according to the morphology of pore structure and the dissolution and removal of materials after electrochemical processing [16,17,18,19,20]. Electrochemical machining technology and theory are improving day by day. For the simulation analysis of electrochemical machining, a large number of scholars have studied and optimized the simulation model [21]. Mesh reconstruction [22,23], influence of process parameters on machining [24,25,26], electrode shape optimization [27], electrochemical machining of mask [28,29], machining stability [30], multi-field coupling analysis [31], etc. For high-temperature resistant material GH 4169, there are few reports on the accuracy of mask-assisted electrochemical machining cooling holes.

In order to improve the problem of uneven electric field distribution at the entrance stage of electrochemical machining of tube electrode and reduce the stray electric field corrosion, a method of electrochemical machining cooling holes with mask was proposed. In order to study the effectiveness of this method, this paper establishes a mask electrochemical machining simulation model to explore the influence of the mask structure on the current density distribution on the surface of the workpiece, the distribution of electric field lines in the processing area and the distribution of current density on the workpiece surface with time were simulated and analyzed under the conditions of no mask and mask. Finally, the forming precision of the two methods is compared.

## 2. Modeling of Electrochemical Machining of Cooling Holes and Process Evaluation Index

### 2.1. Mathematical Model of Anodic Dissolution in Electrochemical Machining

**Figure 1:**
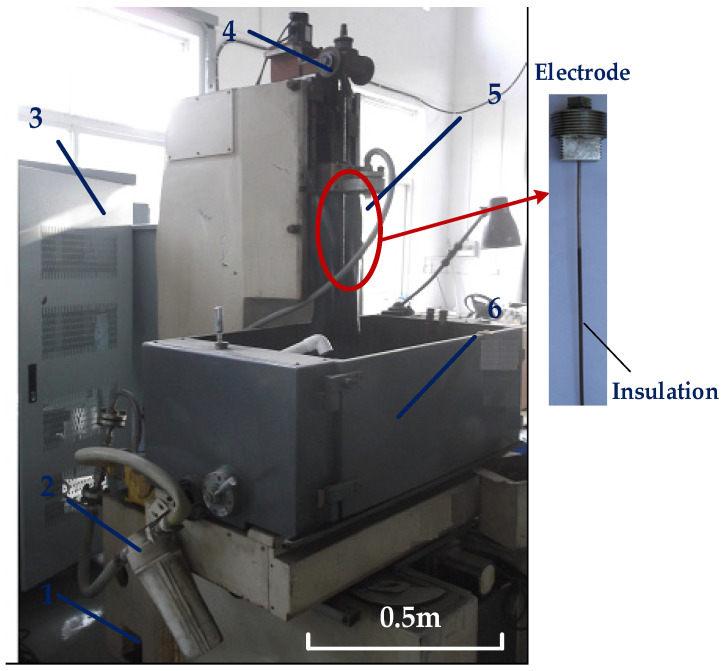
Diagram of ECM equipment processing small hole (1. Filter 2. pump 3. control system 4. motor 5. electrode 6. working table).

The schematic view of the machine and processing system for cooling hole electrochemical machining is shown in Figure 1. There is an insulating layer on the outer surface of the electrode, and the electrolytic reaction occurs only at the end of the electrode. With continuous electrochemical machining, the electrolyte is continuously sprayed from the electrode to the workpiece surface. Electrolysis takes place in the electric field between the tubular electrode and the workpiece. The processing gap is filled with flowing electrolyte, which is the conductor for the electrolytic reaction. Oxidative dissolution occurs near the workpiece, while a reduction reaction occurs near the electrode to produce hydrogen, oxygen, etc. With the continuous addition of new electrolytes, the electrolytic corrosion products and electrolytic pyrolysis in the machining gap are also removed.

The process of electrochemical machining of the mask is as follows. First, coating a layer of liquid photoresist on the surface of the workpiece, and then developing by lithography, the required insulating template with bare structure can be formed on the surface of the workpiece. Next, the hole structure in the non-insulated region of the workpiece is machined by electrolytic machining of the tubular electrode. Among them, the protective effect of the insulating film makes the surface of the workpiece less affected by the current stray corrosion and can be processed into a better size structure. 

In order to establish the mathematical model, the mass chemical equivalent of GH4169 is first determined. The main element composition and content of high temperature resistant nickel-base alloy GH4169 are shown in Table 1. Mass electrochemical equivalent and volume electrochemical equivalent can be calculated by the Equation (1):

(1) $$\begin{array}{l} K=\frac{1}{F\left(\frac{n_{1}}{A_{1}}a_{1}+\frac{n_{2}}{A_{2}}a_{2}+\cdots \cdots +\frac{n_{i}}{A_{i}}a_{i}\right)}\\ \omega =\frac{1}{\rho F\left(\frac{n_{1}}{A_{1}}a_{1}+\frac{n_{2}}{A_{2}}a_{2}+\cdots \cdots +\frac{n_{i}}{A_{i}}a_{i}\right)} \end{array}$$

Substituting the above data into the equation, it can be calculated that the density of GH4169 is about 8.24 g/cm^3^, the volume electrochemical equivalent is about 0.00178 cm^3^/(A∙min), and the mass electrochemical equivalent is 0.01467 g/(A∙min). Based on Faraday’s law, a two-dimensional electric field model of cooling hole in mask electrochemical machining was established. The following assumptions are made for the electric field model:

(1) The influence of temperature and bubble on conductivity is ignored. The conductivity of the electrolyte solution remains constant and isotropic. (2) Electrochemical machining is carried out in an ideal state. That is, the actual value of anode metal dissolution is the same as the theoretical value. The current efficiency η is only related to the current density. (3) The electric field in the processing area is regarded as a stable electric field. The electric field model of machining area is regarded as constant current electric field. The electric field parameter is a function of the relative position. The potential φ(*x*,*y*) at any point in the machining gap between the cathode and anode satisfies the Laplace equation, as shown in the Equation (2):

(2) $$\nabla^{2}\varphi =\frac{\partial^{2}\varphi }{\partial x^{2}}+\frac{\partial^{2}\varphi }{\partial y^{2}}+\frac{\partial^{2}\varphi }{\partial z^{2}}=0$$

An equipotential surface of two different potentials, a constant potential *U* and a ground potential, will form at the interface between the electrode and the electrolyte solution. The insulating template on the surface of the workpiece and the insulating layer on the outer wall of the tube electrode form the insulating boundary. According to Laplace’s equation, the boundary conditions are shown in the Equation (3):

(3) $$\begin{array}{l} Anode\;boundary:\varphi_{a}=U\\ Cathode\;boundary:\varphi_{b}=0\\ Insulating\;boundary:\frac{\partial \varphi }{\partial n}|\Gamma_{c}=0\\ Other\;boundaries:\frac{\partial \varphi }{\partial n}|\Gamma_{s}\approx 0 \end{array}$$

The electric field intensity *E* is the negative gradient of potential *φ*. According to Ohm’s law, the relation between current density *i* and electric field intensity *E* and potential *φ* in the processing area is shown in the Equation (4). Where *E* is the electric field intensity (V/m), *σ* is the conductivity of electrolyte (S/m), and *φ* is the potential (V). The normal dissolution rate of the anode workpiece is shown in Equation (5). By solving the displacement of each point on the workpiece surface with time, the contour morphology of the cooling hole in different times was obtained.

(4) $$\begin{array}{l} E=-\nabla \varphi \\ i=\sigma E=-\sigma \nabla \varphi \end{array}$$

(5) $$v_{n}=\eta \omega i=-\eta \omega \sigma \nabla \varphi$$

### 2.2. Evaluation Index of Electrolytic Machining Cooling Hole Process

**Figure 2:**
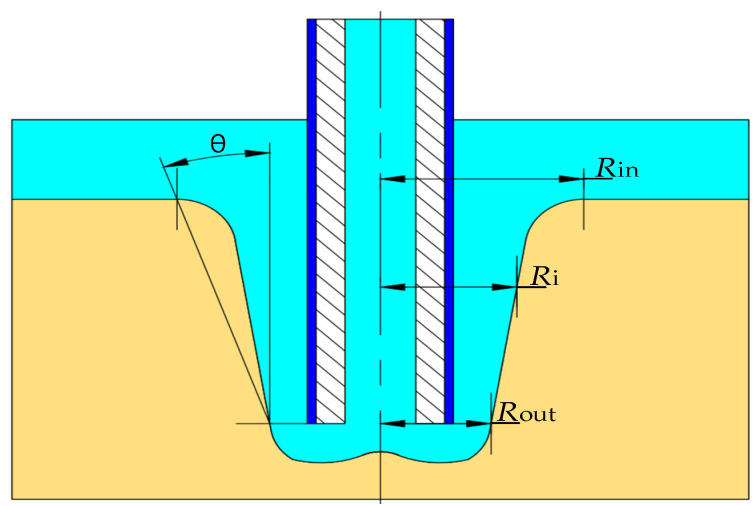
Schematic diagram of electrochemical machining dimensions of cooling holes.

In order to better analyze the forming process of electrolytic machining of cooling holes, the inlet radius (*R*_in_ in Figure 2), outlet radius (*R*_out_ in Figure 2), mean radius (*R*_aver_ is calculated from Equation (6)), taper of electrolytic machining of cooling holes were defined (*θ* in Figure 2), as shown in Figure 2.

The definition of each process evaluation index is as follows:

(1) Mean radius of cooling hole *R*_aver_ (mm), inlet radius *R*_in_ (mm), outlet radius *R*_out_ (mm). The mean radius of the cooling hole is the average from the center line of the tube electrode to the side wall of the hole along the depth direction, which can be obtained by Equation (6):

(6) $$R_{\mathrm{aver}}=\frac{1}{n}\sum_{i=1}^{n}R_{i}$$

(2) Cooling hole taper *θ* (°). Taper refers to the included Angle between the cooling hole side wall obtained by actual electrochemical machining and the theoretical vertical line, and its calculation method is shown in Equation (7). The smaller the taper of the hole, the better the shape accuracy, the more ideal the processing effect. On the contrary, the greater the taper of the side wall of the hole, the worse the shape accuracy, the worse the processing effect. The taper of the side wall of the small hole cannot reach 0°, but it can approach 0° by optimizing the process parameters.

(7) $$\theta =\frac{180}{\pi }\arctan\frac{R_{\mathrm{in}}-R_{\mathrm{out}}}{h}$$

### 2.3. Establishment of Simulation Model of Cool Hole in ECM

**Figure 3:**
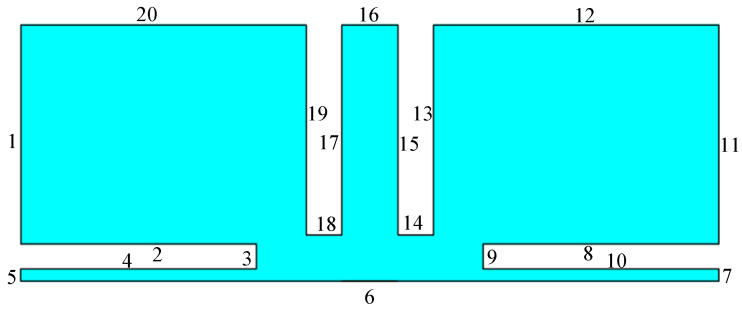
Simulation model of mask electrochemical machining cooling hole.

According to the established electrochemical machining mathematical model and process evaluation standard, the electrochemical machining simulation model of tubular electrode is established. Using the current module and moving grid module of COMSOL Multiphysics finite element analysis software, the formation process of the electric field and size and shape of the cooling holes in mask electrochemical machining are simulated and analyzed. Figure 3 shows a simplified simulation model of cooling holes in mask electrochemical machining. The model was numerically divided into 20 zones. According to Equations (3) and (5), the corresponding electric field conditions are applied to the boundary of the geometric model as shown in Table 2.

The basic simulation parameters of mask electrochemical machining are shown in Table 3. In this paper, 16% NaNO_3_ solution was used as electrolyte, mainly because it is soluble in water and is an ionic compound [32]. The change of flow characteristics can also be ignored, and the dynamic viscosity of water can be used for calculation.

By introducing the principle of electrochemical machining of tubular electrode, the mathematical model of electrochemical machining is established, and the boundary conditions and parameters are set, which provide the support of principle and parameters for simulation modeling and analysis.

## 3. Simulation Analysis

### 3.1. Analysis of Dynamic Forming Process of Cooling Hole

**Figure 4:**
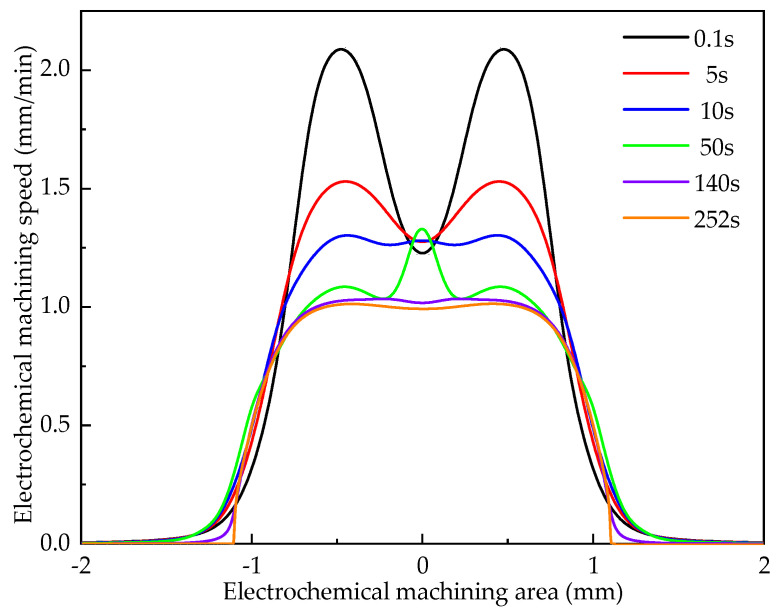
Numerical simulation diagram of cooling hole forming.

In electrochemical machining, under the action of electric field, the surface morphology of workpiece changes due to electrolytic reaction and the geometric structure of machining gap changes. The formation process of cooling holes is numerically simulated by COMSOL software. The conditions used in the simulation are shown in Table 3, acquire the cooling hole forming rule, as shown in Figure 4. As can be seen from the figure, with the increase of processing time, the variation law of cooling aperture accords with Faraday’s law. The forming process of cooling holes can be roughly divided into three stages: 0.1–5 s is the initial stage of machining, and the dissolution rate in the middle area of the workpiece surface is relatively low, while the dissolution rate of the two sides is relatively high; 10–50 s is the middle stage of machining, and the dissolution rate in the middle area of the workpiece surface gradually increases, while the dissolution rate on both sides gradually decreases. The time of 140–252 s is the stable stage of machining, and the dissolution rate of the workpiece surface is relatively uniform.

**Figure 5:**
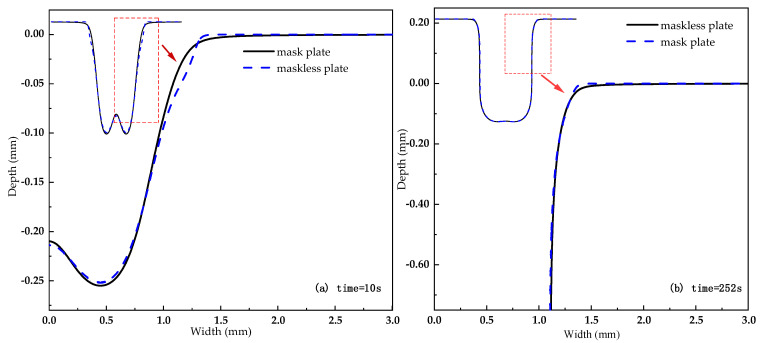
Numerical simulation of the forming of mask and mask-free cooling holes.

Figure 5 shows the numerical simulation diagram of mask and maskless cooling hole formation. It can be seen from Figure 5a that the maskless electrochemical machining has a larger radial overcut at the entrance, a smaller dissolving area on the side wall, and a larger taper of the side wall during processing for 10 s. It can be seen from Figure 5b that as the processing depth increases, the sidewall dissolution range of the two processing methods is basically the same. Therefore, the mask has a greater impact on the accuracy of the ECM entrance, and the impact on the diameter and taper gradually becomes smaller as the processing depth increases.

**Figure 6:**
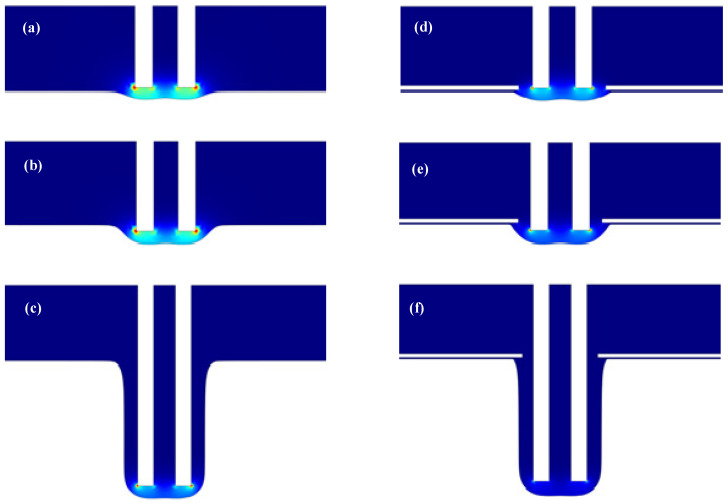
Mask electrochemical machining cooling hole forming process ((**a**–**c**) is the simulation of maskless electrochemical machining process, (**a**) is 10 s, (**b**) is 30 s, (**c**) is 300 s, (**d**–**f**) is the simulation of maskless electrochemical machining process, (**d**) is 10 s, (**e**) is 30 s and (**f**) is 300 s.).

Figure 6 shows the formation process of the cooling hole of the mask electrochemical machining. It can be seen from the figure that the inlet processing stage is 10–30 s, and the diameter change of the cooling hole inlet processing section tends to be stable with the increase of the electrode feed depth. When the processing time is 30–300 s, the diameter change of the ECM cooling hole is gradually stable, and the overall appearance is that the entrance diameter is larger, and the diameter change is smaller during the stable processing stage.

Table 4 shows the data comparison of the forming accuracy of mask electrochemical machining. It can be seen from the table that mask electrochemical machining plays an important role in increasing the entrance radius of the cooling hole. The entrance radius of the mask electrical machining is reduced by about 16.0%, and the end radius and the average radius are increased by about 2.4% and 1.3%, respectively. However, the taper is 52.8% lower, which greatly reduces the taper error of the cooling hole in electrochemical machining.

According to the basic principle of electrochemical machining, under the action of voltage, the electrolyte is sprayed on the workpiece from the inside of the tubular electrode, forming an electric field. Since the mask is insulated, the range of action of the electric field between the electrode and the workpiece is effectively limited [33,34,35]. Therefore, the size and shape of the inlet section can be improved, and the taper of the inlet section due to electrochemical corrosion can be reduced. However, when the machining enters a stable stage, the electric field between the electrode and the workpiece is stable, and the electrolytic reaction speed is balanced with the electrode feeding speed. Therefore, the shapes of the mask stabilizing section are basically the same as that of the unmasked cooling hole machining, and the simulation accords with electrochemical machining.

### 3.2. Simulation Analysis of Electric Field Distribution

**Figure 7:**
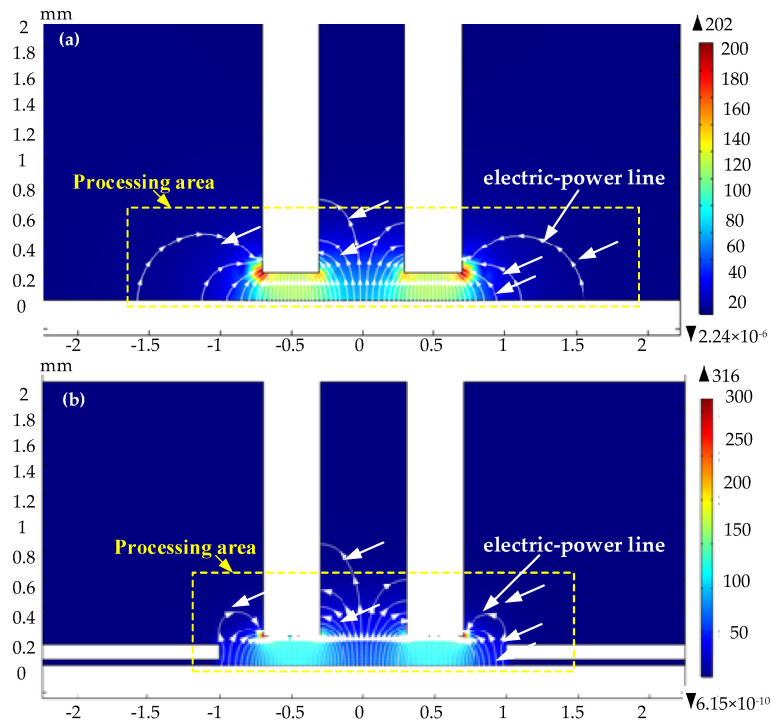
Distribution of electric field lines in the processing area at the initial stage ((**a**) is the simulation of maskless electrochemical machining process and the distribution of power lines, and (**b**) is the simulation of mask electrochemical machining process and the distribution of power lines).

The conditions used in the simulation are shown in Table 3. The mask diameter is 2 mm, and the mask thickness is 0.1 mm. Figure 7 shows the distribution of electric field lines in the machining area at the initial stage of the cooling hole in electrochemical machining. The white lines in the figure are electric field lines. The arrow direction is the direction of current flow. The more concentrated the electric field line, the greater the current density. As shown in Figure 7, the electric field lines are mainly distributed in the machining area composed of electrode end face and workpiece surface. The number of field lines decreases gradually along both sides. Figure 7a the number of electric field lines is 60 and the distribution range is large. Figure 7b the number of electric field lines is 84 and evenly distributed within the mask range. These results indicate that the mask ECM can improve the electric field distribution and the entrance precision of machining.

**Figure 8:**
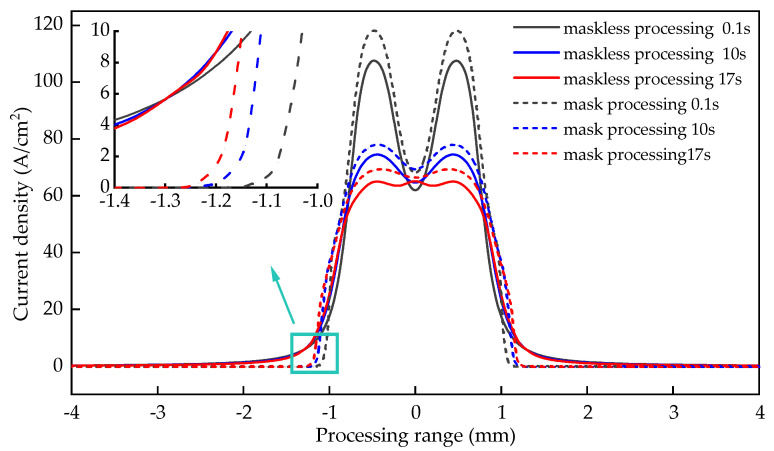
The distribution of current density on the workpiece surface with time in the entrance stage.

The distribution of the surface current density of the workpiece in the entry stage with time is shown in Figure 8. It can be seen from the figure that the current value at the electrode end of the workpiece surface is larger and the distribution range is larger, and it gradually approaches zero along the two sides of the electrode. The surface current density of the mask electrochemical machining workpiece is relatively large. For example, when the machining time is 0.1 s, 10 s, and 17 s, the maximum current density of the mask electrochemical machining at the entrance stage is 118.081 A/cm^2^, 77.917 A/cm^2^ and 69.309 A/cm^2^, the maximum current density of maskless electrochemical machining is 107.307 A/cm^2^, 74.452 A/cm^2^ and 64.933 A/cm^2^, respectively. This shows that the mask electrochemical machining efficiency is higher, the effective current density range (current density >10 A/cm^2^) is more concentrated, and the entrance accuracy is better.

According to Faraday’s law, the greater the number of power lines per unit area, the greater the current density [36,37]. Since the voltage between the electrode and the workpiece is constant, the mask can effectively increase the number of power lines per unit area and the current density between the electrode and the workpiece, so the current density of electrochemical machining with mask is higher than that without mask.

### 3.3. Process Parameter Analysis

In order to better analyze the influence rule of process parameters in the process of ECM, the simulation conditions are as follows: the processing voltage is 24 V, the electrolyte is 16% NaNO_3_ solution, and the inlet flow rate is 8 m/s. The electric field distribution law in the process of cooling hole electrochemical machining is simulated, which provides guidance for analyzing the influence of different technological parameters on the forming law of cooling hole electrochemical machining.

**Figure 9:**
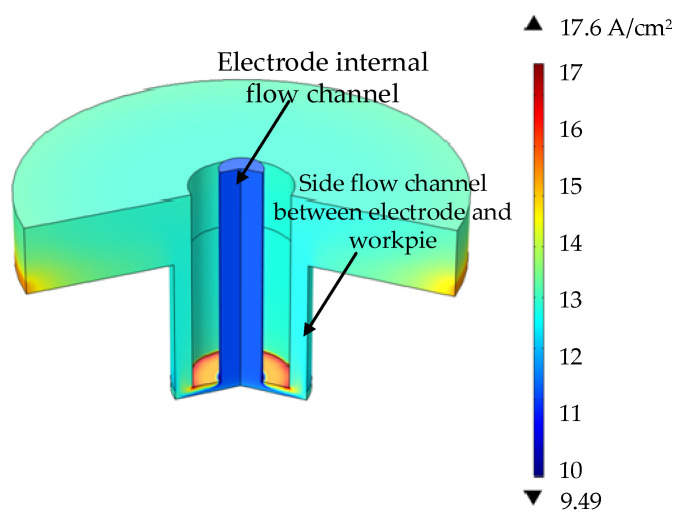
Conductivity distribution in the machining gap.

As can be seen from Figure 9, the minimum conductivity in the machining gap occurs in a narrow area in the end gap, and the maximum conductivity occurs in the corner of the electrode. The electrical conductivity in the gap first decreases and then increases, while the temperature at the side channel and electrolyte outlet gradually increases. The maximum electrical conductivity in the machining gap is 17.6, and the range of electrical conductivity is about 8.1. Main reasons: the electrical conductivity is affected by temperature and gas. When the temperature rises, the electrical conductivity increases, the gas volume fraction increases and the electrical conductivity decreases. At the narrow area in the end face, the temperature and gas volume fraction are particularly small, so the electrical conductivity is the smallest under the combined action of them. The influence of the temperature at the corner of the electrode on the electrical conductivity is far greater than the volume fraction of hydrogen, so the electrical conductivity is the largest.

**Figure 10:**
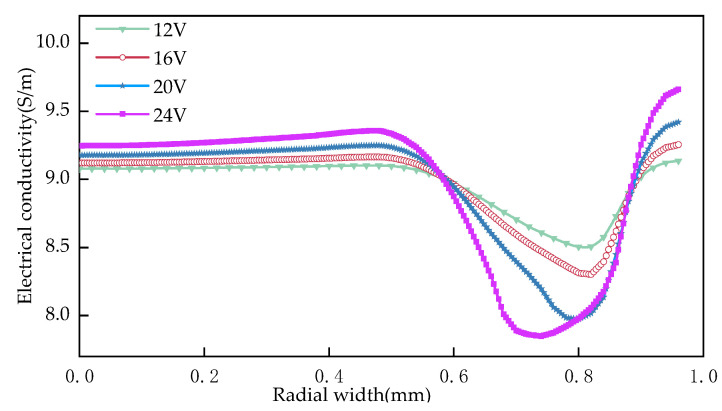
Influence of processing voltage on conductivity distribution.

As can be seen from Figure 10: the conductivity of the front part of electrolyte flow slowly increases with the increase of machining voltage, and the conductivity of the second half decreases rapidly with the increase of machining voltage. When the processing voltages are 12 V, 16 V, 20 V and 24 V, the range of surface conductivity of the workpiece is 0.63 S/m, 0.95 S/m, 1.44 S/m and 1.81 S/m, respectively. The main reason is that the change of electrical conductivity is the result of the interaction of temperature and hydrogen. With the increase of voltage, more and more heat and hydrogen are accumulated in the machining gap, and the increase or decrease of the surface conductivity of theworkpiece is faster and faster with the increase of machining voltage. It can be seen from Table 5 that the average current density of the workpiece surface increases with the increase of voltage, which is beneficial to improving the efficiency of cooling holes in electrochemical machining. However, with the increase of machining voltage, the range and variance of current density on the workpiece surface gradually increase, which leads to “bulge” on the workpiece surface during machining and increases the profile error. 

Different inlet flow rates of electrolyte (6 m/s, 9 m/s, 12 m/s, 15 m/s) were used to simulate and analyze cooling holes in electrochemical machining. Select fixed processing parameters, that is, the processing voltage was 20 V, and the electrolyte was 12% NaNO_3_ solution. The temperature, hydrogen volume fraction and conductivity distribution of the workpiece surface under different electrolyte inlet flow rates are analyzed, and the current density distribution of the workpiece surface to the tool electrode under multi-physical field coupling conditions is studied.

**Figure 11:**
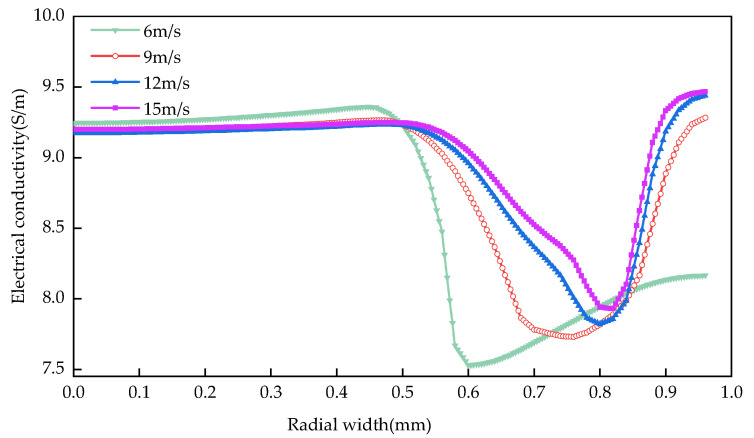
Influence of inlet flow rate on conductivity distribution.

As can be seen from Figure 11: when the inlet flow rate is low, the conductivity of the front part of electrolyte flow increases relatively quickly, and with the increase of inlet flow rate, the conductivity of the second half of the electrolyte flow gradually increases, but generally keeps the law of decreasing first and then increasing. When the inlet flow rate increases within a certain range, the electrical conductivity changes little. When the maximum inlet velocities are 6 m/s, 9 m/s, 12 m/s and 15 m/s, the surface conductivity ranges of the workpiece are 1.831, 1.535, 1.413 and 1.319, respectively. Main reasons: Low-speed electrolyte can not take away heat and hydrogen in the machining gap in time, which leads to the continuous rise of the overall temperature of the workpiece surface and the rapid accumulation of hydrogen. The increase of flow velocity will aggravate the eddy current effect and increase the difficulty of removing heat and hydrogen. The electrical conductivity in the front part increases slowly mainly due to the influence of temperature, while the electrical conductivity of the second half decreases rapidly mainly due to the increase of hydrogen volume fraction, while the electrical conductivity increases continuously with the decrease of hydrogen content and the continuous increase of temperature.

It can be seen from Table 6 that with the increase of electrolyte flow rate, the average range and variance of current density on the workpiece surface gradually decrease, but the overall change range is small. Increasing electrolyte flow rate is beneficial to improve the uniformity of current density on the machined surface of the cooling hole.

According to the mathematical model of electrochemical machining established in Section 2.1, it can be seen that when the voltage between the electrode and the workpiece increases, the current density increases, the electrolyte flow at the inlet of the electrode increases, the removal rate of electrolysis products, electrolysis heat and bubbles between the electrode and the workpiece in unit time increases, the electrolyte renewal rate increases and the current density increases [38,39]. Therefore, the analysis in this section is correct and is consistent with the mathematical model established in Section 2.1.

## 4. Experimental Verification of Electrochemical Machining

In this part, the simulation model analysis is verified by experiments, and the electrochemical machining process parameters and workpieces are the same as the simulation settings. The specific experimental conditions are shown in Table 7. The workpiece is GH 4169, the electrode is a tubular titanium (high current density efficiency and high corrosion resistance) alloy electrode with an inner diameter of 0.6 mm and an outer diameter of 1.4 mm (covered with PTFE insulating film), and the electrolyte is sodium nitrate aqueous solution (according to the workpiece material GH 4169, strong acid electrolyte is selected, which has high current density efficiency).

**Figure 12:**
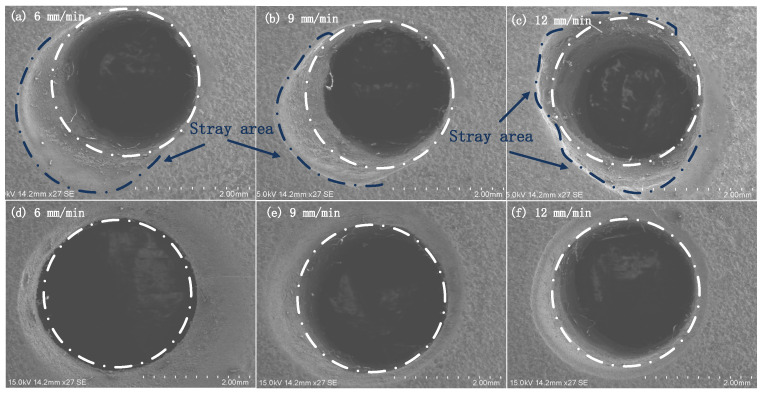
Morphology of cooling hole inlet.

**Figure 13:**
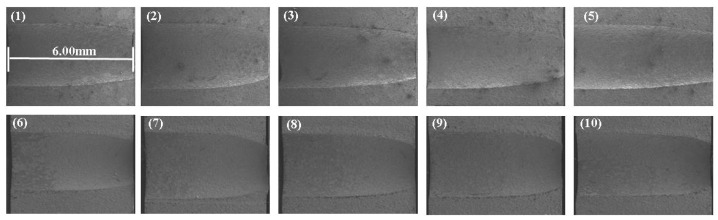
Sectional view of electrochemical machining of cooling holes. (1)Taper 4.40° (2)Taper 4.35° (3)Taper 4.38° (4)Taper 4.41° (5)Taper 4.47° (6)Taper 2.21° (7)Taper 2.64° (8)Taper 2.24° (9)Taper 2.33° (10)Taper 2.52°.

Combined with the parameter values of mask diameter and mask thickness in Table 3 and the parameter values of voltage, electrolyte concentration and electrolyte flow rate in Table 7, an orthogonal test was carried out, and the hole was cut open by wire cutting. According to the Formulas (6) and (7), measure the inlet diameter and calculate the taper of cooling holes, analyze the change of inlet diameter and taper in mask electrochemical machining and maskless electrochemical machining, and analyze the influence rules of different mask diameters and mask thicknesses on the inlet diameter and taper of cooling holes. The entrance morphology and side taper of small holes are measured by SEM, as shown in Figure 12 and Figure 13.

The analysis shows that in maskless electrochemical machining, the range of electric field lines between electrode and workpiece is larger than that in maskless electrochemical machining, which leads to stray corrosion [40,41,42]. As shown in Figure 12a–c, the stray area is irregular, which is related to the uniformity of the composition distribution of GH 4169 alloy. However, in maskless ECM, the stray corrosion at the inlet end is obviously reduced, and the appearance accuracy of the inlet section is high, as shown in Figure 12d–f. The experimental results are consistent with those of the three-part simulation analysis, which proves the authenticity and reliability of the three-part simulation analysis.

The Figure 13 shows the cross-sectional view of the actual machining hole (1–5 mark electrochemical machining and 6–10 mark less electrochemical machining).

As can be seen from Figure 13, when using maskless ECM (1–5), the diameter of the inlet end of ECM is larger than that of the middle section, and the taper of the whole cross section is larger; when using mask ECM (6–10), the diameter of the inlet end of ECM is smaller than that of the middle section, and the taper of the whole cross section is smaller. This is because the mask structure effectively concentrates the electric field area at the entrance end, which is consistent with the simulation results in Section 3.2.

**Figure 14:**
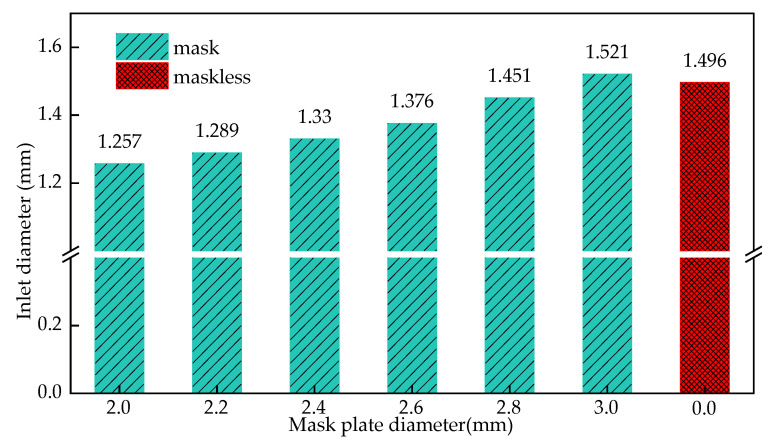
The influence of the mask diameter on the entrance radius of the cooling hole.

Figure 14 shows the influence of the diameter of the mask on the entrance radius of the cooling hole when the height of the mask is 0.1 mm. It can be seen from the figure that as the diameter of the mask increases from 2 mm to 2.8 mm, the entrance radius of the cooling hole increases from 1.257 mm to 1.451 mm, indicating that the improvement effect of the mask structure on the entrance size of the cooling hole increases with the increase of the mask diameter and gradually decreases. For example, when the diameter of the mask continues to increase to 3 mm, the cooling hole entrance radius is 1.521, and the influence of the mask structure disappears.

**Figure 15:**
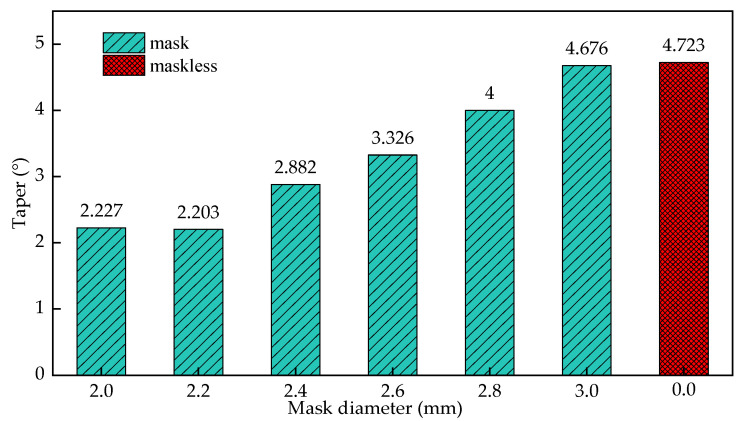
The influence of the diameter of the mask on the taper of the cooling hole.

Figure 15 shows the influence of the mask diameter on the taper of the cooling hole. It can be seen from the figure that as the diameter of the mask increases from 2.0 mm to 3 mm, the taper reaches its minimum value before the diameter of 2.2 mm, and then gradually increases. Therefore, reducing the diameter of the mask does not always reduce the taper of the cooling hole. When the mask diameter is 2.2 mm, the taper of the cooling hole is reduced by 53.4% compared with electrochemical machining, and the improvement effect is the best.

**Figure 16:**
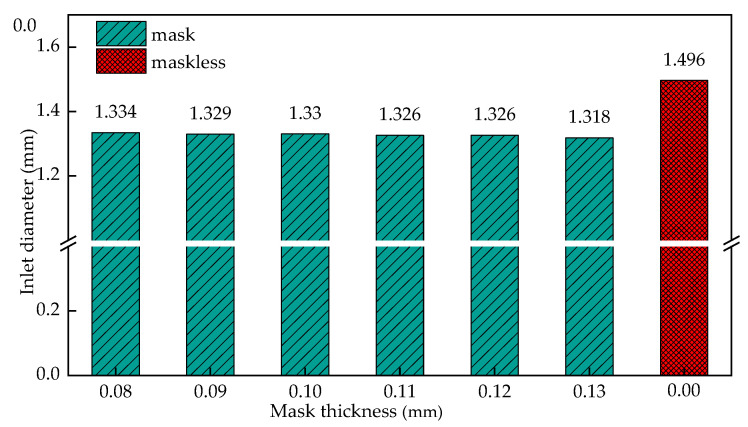
The influence of the mask thickness on the entrance radius of the cooling hole.

Figure 16 shows the influence of the mask thickness on the initial radius of the cooling hole when the mask diameter is 2.2 mm. It can be seen from the figure that when the mask thickness is increased from 0.08 mm to 0.13 mm, the overall cooling hole radius changes little, floating in the range of 1.318 mm to 1.334 mm, but the overall size is lower than the ECM entrance size. It can be seen that the change of the mask thickness has little effect on the distribution of the entrance radius of the cooling hole.

**Figure 17:**
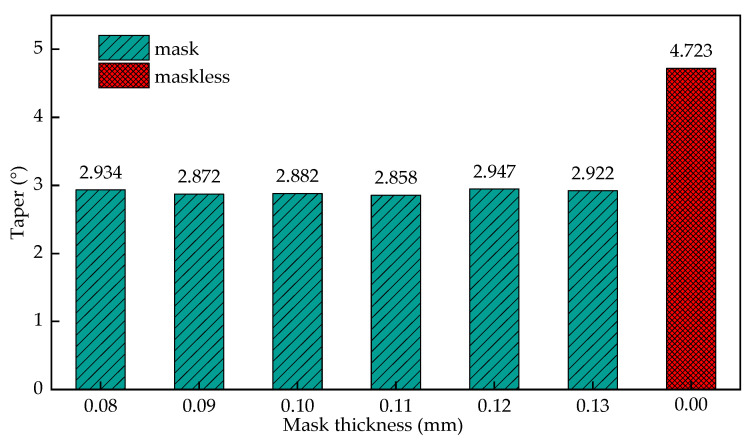
The influence of the mask thickness on the entrance radius of the cooling hole.

Figure 17 shows the effect of mask thickness on the entrance radius of the cooling hole. It can be seen from the figure that when the mask thickness is increased from 0.08 mm to 0.13 mm, the taper of the cooling hole fluctuates in the range of 2.858° to 2.947°, and the overall floating range is small, but the overall taper is lower than the ECM entrance. The change of the mask thickness has little effect on the distribution of the taper of the cooling holes.

Through the comparative analysis of the above experiments, it can be seen that the appearance of the entrance of the mask ECM is good, which is consistent with theoretical model analysis and simulation analysis [43,44]. The structural insulation of the mask can improve the current density between the electrode and the workpiece and reduce stray corrosion, thus verifying the accuracy of the second part of mathematical modeling and the third part of simulation analysis. At the same time, the influence of the thickness and diameter of the mask on the entrance diameter and taper of the cooling hole ECM is analyzed.

## 5. Conclusions

In this paper, through studying the principle of electrochemical corrosion, combining with classical electrochemical theory, the simulation model of mask electrochemical machining is established, and the distribution of electric field in the process of mask and unmasked electrochemical machining is analyzed. Through simulation experiments, the influence of different mask diameters and heights on the size and morphology of cooling holes in electrochemical machining is studied, and the mask electrochemical machining process has no damage and can be reused, which improves the machining efficiency [45,46]. Based on the current density of workpiece surface obtained by multi-physical field coupling simulation model, with the help of any Lagrange-Eulerian formula, the mathematical model of anode boundary movement with respect to current density is established, and the formation law of the size and shape of cooling holes in electrochemical machining under different machining voltages and electrolyte inlet flow rates is analyzed. The conclusions are as follows.

1. Establish the simulation model of cooling hole in mask electrochemical machining. The electric field distribution in the initial stage of masked and unmasked electrochemical machining was compared. The dynamic forming process of cooling hole in electrochemical machining is obtained. the deviation between experimental and simulated inlet diameter is 5.6%, the deviation between experimental and simulated outlet diameter is 5.9%, the deviation between experimental and simulated average diameter is 2.9%, the deviation between experimental and simulated taper is 4.3%.
2. The simulation model of cooling holes in electrochemical machining is established, and the distribution of electrical conductivity in machining gap is adjusted. The relationship between this rule and processing voltage and inlet flow is analyzed by simulation. Simulation result show that the uniformity of electric field distribution is poor with the increase of processing voltage; The current density distribution on the surface of cooling holes under different process parameters is also studied. With the increase of processing voltage from 12 V to 24 V, the surface conductivity of the workpiece increases from 0.63 s/m to 1.81 s/m. When the inlet speed increases from 6 m/s to 15 m/s, the surface conductivity of the workpiece decreases from 1.83 s/m to 1.32 s/m. The results show that the machining voltage is an important factor that affects the machining accuracy of cooling holes, and the influence of the change of inlet speed is relatively small.
3. Under different mask conditions, the size characteristics of cooling holes were analyzed. The results show that the inlet radius of cooling hole decreases with the decrease of mask diameter. The change of mask thickness has little effect on the forming precision of cooling hole. The diameter of the mask increases from 2 mm to 2.8 mm, the entrance radius of the cooling hole increases from 1.257 mm to 1.451 mm, when the diameter of the mask continues to increase to 3 mm, the cooling hole entrance radius is 1.521, and the influence of the mask structure disappears. The diameter of the mask increases from 2.0 mm to 3 mm, the taper reaches its minimum value before the diameter of 2.2 mm, and then gradually increases.

To sum up, mask electrochemical machining can effectively improve the machining accuracy of GH 4169 cooling hole and provide a new machining method for improving the service performance and service life of aero-engine.

## Figures and Tables

**Table 1:** The main elements and content of GH4169.

Element	Ni	Cr	Nb	Mo	Ti	Ai	Fe
Atomic mass	59	52	93	96	47	27	56
Percentage	50–55	17–21	4.75–5.5	2.8–3.3	0.65–1.15	0.2–0.8	Margin

**Table 2:** Boundary condition setting of cooling hole for mask electrochemical machining.

	numerical value	5/6/7	14/15/17/18	2/6/4/8/9/10/13/19
Current module	boundary conditions	electrical potential	electrical potential	electrical isolation
	set	*V* = *U*	*V* = 0	*n*×*j* = 0
	numerical value	5/6/7	13/14/15/17/18/19	Remaining boundary
Mobile grid	boundary conditions	speed	speed	Fixed grid
	set	$V_{n}=\eta \omega i$	$V_{x}=0,V_{y}=v$	

**Table 3:** Basic simulation parameters of mask electrochemical machining.

Basic Parameter	Numerical Value
Voltage(V)	22
Mask diameter (mm)	2/2.2/2.4/2.6/2.8/3
Mask thickness(mm)	0.08/0.09/0.1/0.11/0.12/0.13
Electrode feed rate (mm/min)	0.7
Feed depth (mm)	6.2
Initial clearance (mm)	0.2
Electrolyte conductivity (S/m)	10.1
Initial temperature (K)	293.15

**Table 4:** Comparison of forming accuracy of mask electrochemical machining.

	Entrance Radius R_in_ (mm)	Front Radius R_end_(mm)	Mean Radius Raver (mm)	Taper *θ* (°)
Mask electrochemical machining	1.257	1.024	1.106	2.227
Error (%)	3.9	4.0	3.0	4.0
Maskless electrochemical machining	1.496	1.000	1.092	4.723
Error (%)	5.5	5.7	3.5	4.3

**Table 5:** Analysis of current density on workpiece surface under different machining voltages.

Voltage/V	Average Value/A/cm^2^	Extreme Difference/A/cm^2^	Variance
12	56.64	28.76	11.21
16	76.74	40.05	15.5
20	97.89	53.14	20.53
24	120.62	69.85	26.66

**Table 6:** Analysis of current density on the surface of workpieces atdifferent inlet flow rates.

Flow Rate/m/s	Average Value/A/cm^2^	Extreme Difference/A/cm^2^	Variance
6	100.5	58.6	21.96
9	97.79	53.25	20.67
12	97.06	51.87	20.04
15	96.72	51.17	19.8

**Table 7:** Experimental conditions of electrochemical machining of cooling holes.

Experimental Project	Condition
Electrode	Titanium alloy tube electrode
Workpiece	GH4169 nickel base superalloy
Processing voltage	12–24 V (DC voltage)
Electrolyte concentrations	16% NaNO_3_ solution
Electrolyte flow rate	6–12 mm/min

## Data Availability

Data is contained within the article.

## Abbreviations

ECM	Electrochemical machining
*K*	mass electrochemical equivalent
ω	volume electrochemical equivalent
*A*_1_, *A*_2_,…*A*_*i*_	the relative atomic mass
*n*_1_, *n*_2_,…*n*_*i*_	the valence
*a*_1_, *a*_2_,…*a*_*i*_	percentage of metal element content
*φ*	the potential
*E*	the electric field intensity
*σ*	the conductivity of electrolyte
*R*_in_	the inlet radius
*R*_out_	theoutlet radius
*R*_aver_	themean radius
*θ*	thetaper of electrolytic machining of cooling holes
*n*	the sampling times along the radius of the cooling hole depth
*R*_*i*_	the measured radius along the radius of the cooling hole depth
